# The relationship between meat disgust and meat avoidance—A chicken-and-egg problem

**DOI:** 10.3389/fnut.2022.958248

**Published:** 2022-09-02

**Authors:** Elisa Becker, Stella Kozmér, Matthias B. Aulbach, Natalia S. Lawrence

**Affiliations:** ^1^Psychology Department, University of Exeter, Exeter, United Kingdom; ^2^Centre for Cognitive Neuroscience, Department of Psychology, University of Salzburg, Salzburg, Austria; ^3^School of Science, Aalto University, Espoo, Finland

**Keywords:** meat disgust, meat avoidance, evolution, vegetarian diet, eating behavior

## Abstract

Feelings of disgust toward meat have been researched for at least 30 years, but so far the causal relationship that may link meat disgust and meat consumption has remained elusive. Two possible pathways have been proposed in previous literature: the more common pathway seems to be that meat disgust is developed after a transition to vegetarianism, potentially *via* the process of moralization and recruitment of (moral) disgust. Other accounts suggest the existence of a second pathway in which disgust initiates the avoidance of meat and this can be explained by existing theories of disgust functioning as a pathogen avoidance mechanism and meat serving as a pathogen cue. However, the evidence base for either relationship remains thin and to our knowledge no research has examined whether temporary meat abstention can lead to increases in meat disgust, as the first pathway suggests. We measured meat disgust and meat intake in *n* = 40 meat eaters before and after attempting a meat-free diet for 1 month (while taking part in the annual vegan campaign Veganuary). Although most participants lapsed to eating meat during this period, we found that reductions in meat intake during the month were predictive of increases in meat disgust afterwards. This supports the view that meat disgust is expressed as a result of meat avoidance in meat eaters. Implications for theoretical understanding of the relationship between meat disgust and meat avoidance, as well as the development of disgust based interventions are discussed.

## Introduction

Meat production is an inefficient and unsustainable way of feeding the world's population (1), and is the cause of animal suffering (2). Many UK consumers are aware of these negative impacts of meat production and up to a third contemplate reducing their meat intake (3, 4), but perceptions of meat being “normal,” “necessary,” “natural,” and “nice” means many people find it hard to resist (5–7). On the other hand, feelings of disgust toward meat have been well-documented in those who do not consume it [i.e., vegetarians and vegans (8–10)]. We define as “meat disgust” only those disgust responses toward meat that is cooked and/or ready for consumption, unspoilt, and culturally appropriate to eat. Not surprisingly, in vegetarians, feelings of meat disgust predict stricter adherence to a vegetarian diet and fewer lapses (11). However, meat disgust is not strictly a vegetarian phenomenon—disgust toward red meat in particular has been frequently reported among omnivores (10, 12, 13), and there is evidence that meat disgust is a good predictor of lower meat consumption in omnivores and flexitarians (14). It seems that meat disgust could be a powerful factor counteracting meat liking and temptation and could help people reduce their meat intake and transition to a meat-free diet. However, we still know very little about this relationship, including any causal mechanisms.

The present paper aims to address this gap by first reviewing existing evidence for different causal relationships between meat disgust and meat intake (or rather, avoidance), and secondly, presenting a study of meat eaters that reduced or eliminated their meat intake for one month while taking part in the Veganuary campaign—a pledge to follow a vegan diet for the duration of January (www.veganuary.com) (15)—allowing us to observe if any changes in meat disgust followed this diet change or whether baseline disgust prior to participation in Veganuary predicted successful meat avoidance. Establishing a temporal order will bring us one step closer to understanding the mechanism that links meat disgust and meat avoidance and could enable us to harness the power of disgust in interventions that initiate or maintain the practice of meat avoidance.

### Literature review

The relationship between meat disgust and meat consumption is still not well understood. Most existing studies of meat disgust have thus far focused on vegetarians who were already meat disgusted (8, 9, 16, 17), and this makes it difficult to draw conclusions about any causal relationship between meat disgust and meat avoidance (i.e., whether meat disgust is present first and causes meat avoidance or whether people decide to become vegetarians and develop meat disgust later). However, there is some evidence on the temporal order and causal relationship between the two (mostly from the accounts that vegetarians have given on how the relationship had developed in themselves), which will be reviewed in this part of the paper. While the evidence discussed below has large gaps (for instance, there is no evidence on the role of meat disgust in starting a meat-reduced diet rather than a vegetarian diet) it is still helpful in testing the plausibility of the several possible temporal and causal relationships that could link meat disgust and meat avoidance.

One possible mechanism that may link meat disgust and meat intake is that meat disgust develops after people have stopped (or reduced) their meat intake. Evidence for this possibility comes from a study by Paul Rozin et al. (17) who asked vegetarians to indicate their agreement with 20 different reasons for following a vegetarian diet, including feelings of disgust toward meat. Additionally, they asked their participants to indicate whether each of those reasons had been a reason for transitioning to vegetarianism in the first place. It is interesting to see that although 53% of their 104 subjects agreed or strongly agreed that they currently avoid meat because they find it disgusting, only 14.4% said that feelings of disgust toward meat had been an initial reason that led them to start following a vegetarian diet. Rozin et al.'s (17) findings therefore suggest that in the majority of vegetarians who feel disgusted by meat, these feelings developed after the transition to vegetarianism. This order of events is also corroborated by a number of qualitative studies (8, 9, 17–19) in which vegetarians report that they chose to stop eating meat for various reasons but are now (at the point of interviewing, often years after the transition) so disgusted by meat that they cannot bring themselves to eat it, not even to avoid conflict or embarrassment when they are served meat in a social situation (19). This demonstrates how the expression of disgust after the transition to vegetarianism would serve to maintain the practice of meat avoidance without the need for self-control.

One mechanism that has been proposed for the recruitment of disgust after transitioning to vegetarianism is moralization (17). According to this theory, activities that were previously perceived as morally neutral can acquire moral significance which then enables the development of norms and values about that activity. Meat consumption, the authors claim, is such an activity and once transitioned to vegetarianism, some people start to moralize and condemn meat consumption which turns their preference for vegetarianism into a value and part of their identity (17). One part of the moralization process seems to be the recruitment of disgust, which can be a moral emotion (20). Many researchers have reported that feelings of disgust toward meat are more common among those who follow a vegetarian diet for moral reasons [and have possibly, according to Rozin et al. (17) gone through a process of moralization] than those who do so for health reasons (e.g., 11, 17, 21). However, a longitudinal study by Feinberg et al. (21) demonstrates that moralization of meat eating can occur in omnivores before (or indeed without) a transition to vegetarianism. These authors suggest that the experience of meat disgust serves as a conduit for moralization, thereby turning the causal relationship proposed by Rozin et al. (17) on its head, which is also supported by others (13). Therefore, the moralization process does not seem sufficient in explaining the temporal or causal order of meat disgust and meat avoidance. In any case, moralization can only explain some cases of meat disgust, namely those in which moral disgust toward meat is experienced, rather than other, more basic forms like core disgust (22, 23).

The same studies that show that meat disgust commonly follows meat avoidance also deliver evidence for the reverse pathway—meat disgust causing meat avoidance. For instance, a minority (14.4%) of the vegetarians in Rozin et al.'s (17) study (described above) reported that meat disgust caused them to stop eating meat. Confirming this, other studies also cite vegetarians who give disgust as the reason for giving up meat, and then usually as the result of a single disturbing and often disgusting “conversion experience” (9) like this interviewee describes:

“I was cooking breakfast which was a cup of tea and a bacon cob . . . And that morning the smell of bacon was quite off-putting... And then, I was eating the cob, and I'd just taken a bite of it... and then, the next bite, the rind wasn't cooked properly. And the rind stayed in my mouth, and came off the meat, and sort of dangled from—from the corner of my mouth. And I—heaved, and put the cob down, and that was the end.” (9)

Similar accounts of emotionally upsetting experiences (often also combined with guilt and sadness, rather than pure disgust) leading people to spontaneously become meat-disgusted vegetarians can be found elsewhere [e.g., (8)].

Even without a moral component, meat seems to elicit feelings of disgust much more easily than any other food: for example, pairing meat images with disgust stimuli in an evaluative conditioning experiment reduced willingness to eat meat, while the same disgust conditioning was ineffective for vegetables and beverages (24). Similarly, presenting meat dishes with a label that makes them seem unfamiliar (e.g., presenting a beef steak as “langua steak”—a fictitious animal name) can elicit disgust in prospective eaters, whereas plant-based foods with the same treatment do not (25). Tybur et al. (26) propose a possible mechanism for this phenomenon. Based on the widely accepted theory that disgust has evolved as a behavioral pathogen avoidance system (27–30), these authors suggest that objects that pose pathogen risks (such as meat—in essence a corpse that will soon rot) serve as inputs or heuristic cues for a risk-benefit computation, one output of which is a disgust response (26). This “preparedness” (31) for meat to be perceived as disgusting could explain why some people suddenly get disgusted by meat, to the point where it leads them to stop eating it.

To further complicate things, it should be noted that neither meat disgust nor meat consumption are binary “on/off” concepts but both exist and affect each other along a continuum as we have shown in a previous study (14). We quantified levels of meat disgust in vegetarians, flexitarians, and omnivores using visual analog scale ratings of images of meat that had elsewhere been rated as highly palatable (32). While on average, levels of meat disgust were highest in the vegetarian sample, flexitarians and even some omnivores also expressed some levels of disgust toward meat which was consistently higher than disgust toward control images of carbohydrate-rich (plant-based) foods. Using individual levels of meat disgust to predict individual levels of meat intake in omnivores and flexitarians, we found meat disgust to be the strongest predictor of reduced meat intake in flexitarians (whereas surprisingly self-control was not predictive of meat intake) and omnivores (here only after controlling for participant age). Furthermore, we found that naturally occurring decreases in meat intake over 6 months (in the absence of interventions) were associated with increases in meat disgust in both of these diet groups (14). This demonstrates that people cannot be classed as either meat disgusted or not, but that instead, meat disgust is experienced by everyone to varying degrees and is associated with how much meat a person consumes. Seeing meat disgust (and meat intake) as continuous variables has important consequences for research questions about this relationship, and rather than asking “which came first?,” it might be more appropriate to ask whether increases in meat disgust can result from decreases in meat intake. This is what the current study aims to test.

### The present study

In summary, there is good theoretical grounding and evidence for both accounts of the meat-disgust-meat-avoidance relationship (i.e., meat disgust causing meat avoidance; or meat avoidance leading to increased meat disgust). Other studies have already delivered some evidence that increasing disgust to meat can lead to reduced consumption of or willingness to eat meat (24, 25, 33, 34) but the reverse pathway (meat avoidance leading to meat disgust) needs more quantitative evidence. This study aims to test one direction of the possible causal relationship and asks whether temporary decreases in meat intake can result in increases in meat disgust.

An ideal study design to test the effect of reduced meat intake on meat disgust would randomly assign participants to conditions in which they have to either avoid meat consumption or not. At the time of data collection, we assumed that this would be very hard to recruit for (other than with large participant payments) and even harder to affirm that any reductions in meat intake had actually taken place [although recent research has achieved this *via* the use of daily smart-phone surveys (35)]. Instead, we propose that the annual health challenge of “Veganuary” (www.veganuary.com) presents an ideal opportunity for a field study on meat avoidance. We surveyed people that planned to take part in Veganuary of their own accord, and, at the time of the survey still ate at least some meat. Their meat disgust and meat intake levels were measured at baseline (pre-Veganuary), along with other factors that potentially affect goal-directed eating behavior and disgust. At follow-up (post-Veganuary), the participants were asked how much meat they had consumed during Veganuary and their meat disgust levels were measured again.

From our previous investigation of the link between meat disgust and meat intake (14), we know that even in omnivores and flexitarians (who constituted the sample of the present study) meat disgust predicts some variance in meat intake. We expected this relationship to be replicated in the present study, at baseline, before people embarked on the Veganuary challenge. In line with the majority of evidence presented in the literature review, we further predicted that a 1 month period of meat avoidance would lead to increases in meat disgust. The present study therefore tested the following pre-registered hypotheses^*^:

H1: Meat consumption will be negatively associated with meat disgust at baseline.
H2: Any decreases in meat consumption during Veganuary will be associated with increases in meat disgust at follow-up.

Additionally, we also explored factors that may explain better adherence to a meat-free/meat-reduced diet during Veganuary using a measure of restrained eating in a quantitative analysis, as well as qualitatively analyzing participants' own comments. While this is not the focus of the current study it may add to the research on barriers to and facilitators of transitions to meat-free diets and should further help to embed the role of disgust within a wider range of factors at play.

^*^Note: Slightly different hypotheses were pre-registered at https://aspredicted.org/ZBK_Y37 for H1 and H2 but had to be amended because the original hypotheses had rested on the assumption that most or all of the participants would follow a meat-free diet during Veganuary (as that was their intention at the time of pre-screening). However, only 11 participants followed a completely meat-free diet during Veganuary. For this reason, two of the three pre-registered hypotheses (H1: “Eliminating meat consumption for one month will be associated with increases in meat disgust.” and H3: “In people who did not stick to a meat-free diet, the change in meat intake from baseline to follow-up will be associated with changes in meat disgust over that time.”) were merged into the new H2 presented here.

## Methods

### Participant recruitment and sample

Participant recruitment took place on Prolific (www.prolific.co) (36). In order to gain access to a sample of participants who intended to not eat meat for the duration of January (but still ate at least some meat at the time of baseline recruitment), we conducted a pre-screen survey. This survey was advertised as a survey about health campaigns in January and asked participants to indicate if they intended to take part in any health challenges in January (with options including “Veganuary,” but also other, non-meat related options like “Dry January”). Participants were then asked to identify their current diet on a spectrum from omnivore to vegan and were thanked for their participation. From the responses to this pre-screen we selected only those participants who had reported an intention to take part in Veganuary and reported to be either omnivore or flexitarian.

The pre-screen survey was completed by 1,125 people on Prolific. Of those, 60 were eligible participants (who were either omnivores or flexitarians and intended to take part in Veganuary or a different meat-free January challenge) and were invited to the baseline survey. 48 participants completed this survey without failing more than one of three attention check questions. All of these were invited to the follow-up survey in February which was completed without more than one failed attention check by all of the 40 people who participated in it. Table 1 shows descriptive statistics of our sample.

**Table 1:** Sample characteristics at baseline.

	**Minimum**	**Maximum**	**Mean**	**SD**
Age	19.00	70.00	30.28	11.67
Female gender (%)			82.50	
Disgust sensitivity	0.84	3.68	2.49	0.70
TFEQ scores				
Cognitive restraint	1.17	3.67	2.20	0.61
Uncontrolled eating	1.11	3.67	2.38	0.58
Emotional eating	1.00	4.00	2.47	0.88
Meat intake	0.00	13.00	5.28	2.92
Explicit meat disgust	0.00	85.67	44.64	24.11
Implicit meat disgust	−0.85	1.13	0.27	0.47

### Procedure

The main study was hosted on Qualtrics (37) in December 2020 (baseline) and February 2021 (follow-up). Baseline and follow-up survey both measured the same concepts in identical order: After participants gave informed consent to take part, they completed a short demographics questionnaire [age, gender, country of residence, and hunger level which has previously been shown to affect food disgust ratings (38)]. Participants were also asked to report any existing medical dietary restrictions they may have so that we could control for any added difficulty of restricting an already restricted diet but no participants reported any restrictions.

Following this, we measured meat disgust in two different ways: first, an Implicit Associations Test [IAT (34, 35)] was used to measure meat disgust indirectly in order to avoid self-report biases. Then, disgust to meat and to carbohydrates was measured explicitly using visual analog scales (see Measures section below for more details). The meat and carb images presented as stimuli in the IAT and the VAS were the same, and the IAT was always conducted first so that any anchoring effects from the rating task would be avoided. After the meat disgust measures, the Three Factor Eating Questionnaire (39) and the Disgust Scale—Revised (40) were then used to measure other factors that may affect meat intake and/or disgust, followed by a food frequency questionnaire assessing meat intake.

Additionally, at follow-up, participants were further asked about their intended diet going forward as well as their reasons for taking part in Veganuary. They were given one open-ended question about factors helping them during Veganuary (if they had reported zero meat intake during Veganuary) or factors that made following a meat-free diet harder (if they had reported some meat intake during Veganuary).

### Measures

#### Implicit meat disgust

**Figure 1:**
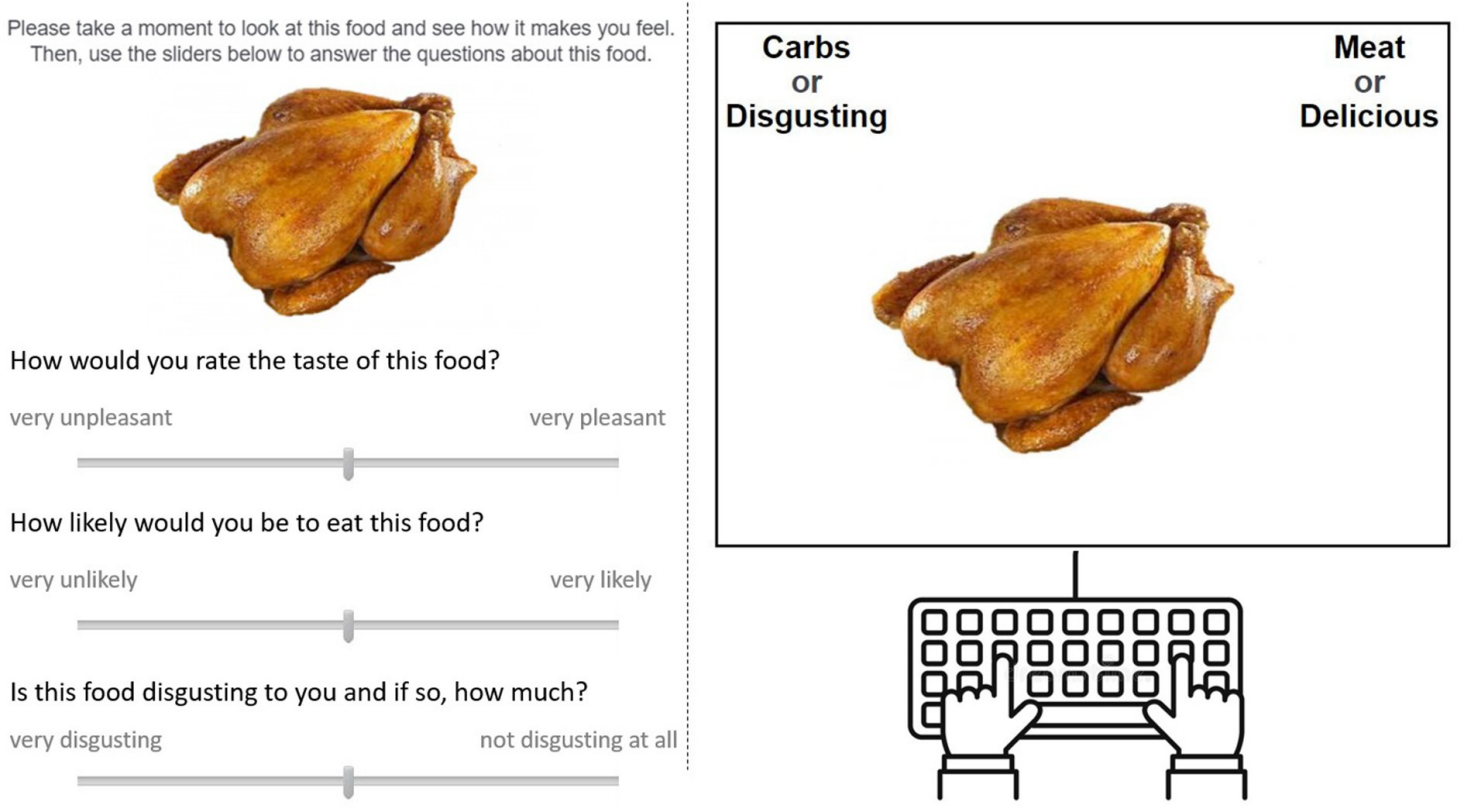
Example trials from the VAS meat/carb rating task (left) and the IAT (right). In both tasks, the same set of images was used (six of meat and six of carbohydrate rich foods).

To test the strength of implicit associations between “meat” and “disgusting”/”delicious” participants performed an implicit associations test (IAT) with the concepts “meat” and “carbs” represented by six images each of meat and carbohydrate-rich foods, respectively, and the attributes “disgusting” and “delicious” represented by synonyms of these words (Figure 1). The IAT was identical to the one used in our previous study (14), with a procedure as described in Greenwald et al. (41). IAT block order was counterbalanced across participants at baseline, and, to avoid order effects (42) that would make the comparison of baseline and follow-up results difficult, we gave each participant the same version of the IAT at follow-up (with the same block order) that they had completed at baseline. The resulting *d*-score from the IAT was reversed to make interpretation more intuitive (stronger positive values indicating stronger bias toward the concept-attribute pairing of “meat” and “disgusting”) and this variable is called “implicit meat disgust.”

#### Explicit meat and carb disgust and liking

Participants were asked to rate six meat and six carbohydrate images (the same ones they had seen in the IAT) on 100-point visual analog scales (VAS) in terms of the taste of the food (from very unpleasant to very pleasant), the likelihood that they would eat the food (from very unlikely to very likely, responses to this question were not analyzed in this study), and the disgustingness of the food (from very disgusting to not at all disgusting). Using images of cooked, unspoilt, and culturally familiar meat including red and white meat, this measure captures specific disgust to “normal” meat that many other people would find appetizing. As such it measures the conceptualization of “meat disgust” that is used in this study and should not be affected by disgust toward signs of spoilage or unusual and unpleasant aspects of meat, as measured by other validated scales such as the Food Disgust Scale (43). Participants were always asked to rate the taste first in an effort to (a) mask the study's focus on disgust and (b) get participants to think about distaste and disgust as two separate concepts (see Figure 1).

#### Eating patterns

In order to control for other factors that may affect how successful participants were at reducing/eliminating meat intake during Veganuary, the 18-item version of the TFEQ was used to assess three dimensions of eating behavior—cognitive restraint, uncontrolled eating, and emotional eating (39).

#### Disgust sensitivity

Participants also completed the Disgust Scale—Revised [DS-R (40)] to assess disgust sensitivity in order to control for any observed changes in meat disgust being driven by this trait. Note that there is also a more specific Food Disgust Scale available (43), however, we opted for a more general scale to measure disgust sensitivity in order to avoid any circularity in the interpretation of any effects of disgust sensitivity on meat disgust (especially since the Food Disgust Scale has a “meat” subscale and therefore correlating this measure with our measure of meat disgust would be somewhat circular).

#### Meat intake

This was assessed by a Food Frequency Questionnaire (FFQ) asking participants how often they consumed various meat items in a typical month (at baseline) or during Veganuary (at follow-up in February), with response options on a seven-point scale ranging from “twice a day or more” down to “1–3 times a month” and “less often or never” [adapted from Churchill and Jessop (44) and Lawrence et al. (45)]. We also assessed consumption of fish and seafood, dairy, and eggs but did not include these in the analysis because this study and our disgust measures focused on meat.

### Stimuli

The stimuli used in this study were the same as in our previous study (14): Six images each of meats and carbs were used in both the implicit and explicit measures (IAT and VAS) and were taken from the *food-pics* database (32) with the exception of one picture of bacon which was not available in *food-pics* and was taken from the internet. The images (see Supplementary Figure S1) were chosen to represent a variety of meats (two each of pork, beef, and chicken) that are commonly eaten in the UK and had high palatability ratings [ratings are available in the *food-pics* database (32)]. The carb images similarly represented different types of carbohydrates (bread, potatoes, rice) and were chosen to visually match the fat content and portion size of the meat images. All images were of foods that were deemed culturally familiar to our UK participants, cooked, and unspoilt.

## Results

Hunger level did not correlate with explicit or implicit meat disgust and was therefore not controlled for in further analyses.

IAT scores may reflect/can be driven by any concept-attribute pairings that are presented within the IAT (in this case meat + disgusting/delicious, carbs + disgusting/delicious). To test that our implicit measure was a reflection of meat disgust and not the other concept associations that contributed to the IAT score, we correlated the IAT scores with the explicit measures of meat disgust, carb disgust, and carb liking. IAT scores showed a marginally significant weak correlation [*r*(40) = 0.283, *p* = 0.077, 95% *CI* (−0.031, 0.547)] with our explicit measure of meat disgust. None of the other explicit measures correlated significantly with the IAT scores, indicating that the IAT scores are most likely a reflection of meat disgust. However, the weak and not very significant correlation are cause for concern and readers should bear this in mind when interpreting the results presented below.

### Hypothesis testing

We first analyzed the relationship of meat disgust and meat intake at baseline (H1: Meat consumption will be negatively associated with meat disgust at baseline) in a regression model. For this model, the six predictor variables age, gender, disgust sensitivity, cognitive restraint, implicit meat disgust and explicit meat disgust were pre-registered for purposes of comparability with Becker and Lawrence (14) where similar predictors were used to predict meat intake. However, because of the low sample size in this study (*n* = 40), the number of predictors should ideally not exceed four in order to still achieve sufficient power to detect at least large effect sizes (46). An alternative model with four predictors can be viewed in the Supplementary material, although there were no qualitative changes in the results other than the observed effect of implicit meat disgust decreasing in size and falling slightly below significance level.

The only significant predictors for meat intake were implicit and explicit meat disgust (Table 2). Interestingly, only explicit meat disgust predicted meat intake in the expected direction (the higher the level of meat disgust, the lower the meat intake). Implicit meat disgust on the other hand had a positive effect on meat intake (the higher the implicit meat disgust, the higher the meat intake at baseline).

**Table 2:** Coefficients from multiple regression on baseline meat intake with six predictors.

	**β**	**95%** ***CI***		***p***
		**Lower**	**Upper**	
Age	−0.242	−0.513	0.029	0.078
Female gender	−0.046	−0.337	0.245	0.752
Disgust sensitivity	0.18	−0.103	0.463	0.205
Cognitive restraint	0	−0.266	0.266	0.997
Implicit meat disgust (T1)	**0.325**	0.047	0.603	0.023
Explicit meat disgust (T1)	**−0.773**	−1.09	−0.455	<0.001

n = 40, R^2^ = 0.481, R${}_{adj}^{2}$ = 0.387. Bold value indicates significant coefficients at *p* < 0.05.

To test H2 (any decreases in meat consumption during Veganuary will be associated with increases in meat disgust at follow-up), we tested whether changes in meat disgust from pre- to post-Veganuary were correlated with changes in meat intake in simple bivariate correlations. Change scores were calculated by subtracting the baseline score from the follow-up score, so that a positive change score would indicate increases in meat disgust or meat intake and *vice versa*.

**Figure 2:**
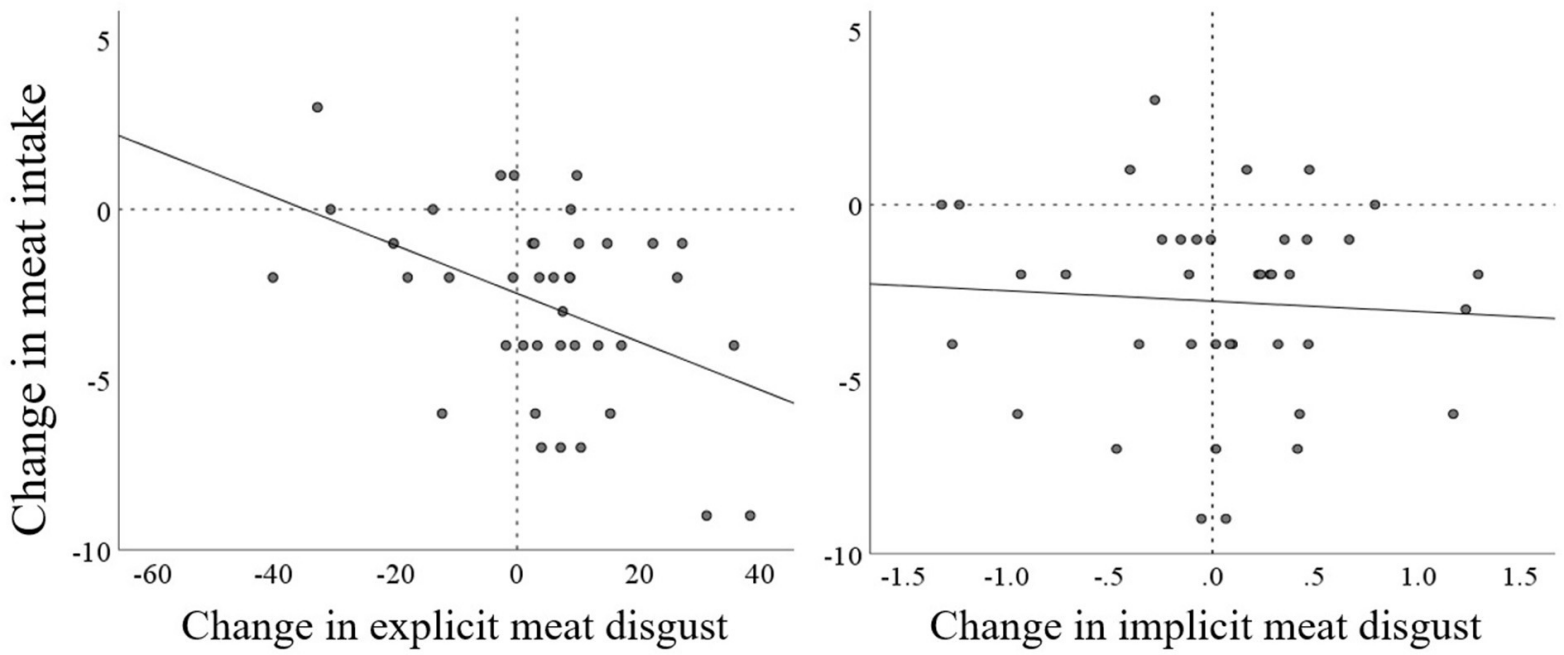
Scatterplot of changes in meat intake as a function of changes in explicit (left) and implicit (right) meat disgust.

Changes in meat intake and changes in explicit meat disgust were negatively correlated in the expected direction [*r*(40) = −0.44^**^, *p* = 0.005, 95% *CI* (−0.661, −0.148)]. For changes in implicit meat disgust there was no significant correlation with change in meat intake [*r*(40) = −0.067, *p* = 0.683, 95% *CI* (−0.37, 0.25)]. Figure 2 shows the relationship of these change scores.

Because changes in explicit meat disgust were associated with changes in meat intake in bivariate correlations, we chose to explore this relationship further by running a regression model to explain the changes in explicit meat disgust using additional predictors (Table 3). As mentioned above, because of the low sample size in this study (*n* = 40), the number of predictors was limited to no more than four in order to still achieve sufficient power to detect at least large effect sizes (46). We chose meat intake change, implicit meat disgust change, cognitive restraint [again, for comparability with our previous study (14) where self-control was included in the model predicting meat disgust], and disgust sensitivity (DS-R) as the most interesting predictors. Age and gender were not tested as predictors because although they might have an impact on the expression of meat disgust, this paper focuses more on psychological as opposed to demographic factors.

**Table 3:** Coefficients from multiple regression on changes in (explicit) meat disgust.

	**β**	**95%** ***CI***		***p***
		**Lower**	**Upper**	
Change in meat intake	**−0.505**	−0.787	−0.223	0.001
Change in implicit meat disgust	0.178	−0.104	0.461	0.208
Cognitive restraint	**0.393**	0.103	0.684	0.009
Disgust sensitivity	−0.141	−0.433	0.150	0.332

*n* =40, *R*^2^ = 0.365, *R*${}_{adj}^{2}$ = 0.293. Bold value indicates significant coefficients at *p* < 0.05.

Changes in explicit meat disgust were significantly predicted by changes in meat intake during Veganuary in the expected direction: the more negative the change in meat intake (i.e., the larger the reduction) the larger the increase in meat disgust. The other significant predictor of changes in meat disgust was restrained eating, such that individuals with higher cognitive restraint (measured at baseline) showed larger increases in meat disgust during Veganuary.

In order to estimate the sensitivity of our regression models to detect different effect sizes, we conducted a *post-hoc* power analysis in G^*^Power (47), estimating the achieved power for single regression coefficients. For small effects (partial *R*^2^ = 0.02/*f*^2^ ~ 0.02), achieved power was 0.14; for medium effects (partial *R*^2^ = 0.13/*f*^2^ ~ 0.15) achieved power was 0.66; and for large effects (partial *R*^2^ = 0.26/*f*^2^ ~ 0.35) achieved power was 0.95.

### Exploratory analyses of additional factors affecting reduced meat intake

Two separate analyses were used to explore the role of other factors in the adherence to a temporary meat-free diet. The first was a final regression model on meat intake during Veganuary, and the second was a thematic analysis of responses to an open question probing factors that affected successful meat avoidance during Veganuary.

The regression on meat intake during Veganuary was run using baseline meat intake, baseline explicit and implicit meat disgust, and cognitive restraint as predictors. After controlling for baseline meat intake, no significant predictors remained in the model (Table 4).

**Table 4:** Coefficients from multiple regression on meat intake during Veganuary.

	**β**	**95%** ***CI***		***p***
		**Lower**	**Upper**	
Meat intake (T1)	**0.579**	0.213	0.945	0.003
Explicit meat disgust (T1)	0.149	−0.235	0.533	0.436
Implicit meat disgust (T1)	0.038	−0.275	0.351	0.806
Cognitive restraint	0.225	−0.062	0.513	0.121

n = 40, R^2^ = 0.316, R${}_{adj}^{2}$ = 0.237. Bold value indicates significant coefficients at *p* < 0.05.

Because all factors of the TFEQ were administered in the survey, and not much is known about these factors and their link to reducing meat consumption, all three factors were in turn used as predictors in all of the above regression models (with emotional or uncontrolled eating replacing cognitive restraint). The results can be viewed in the Table S2, S3 in Supplementary material (only emotional eating was a positive predictor of meat intake during Veganuary).

All 40 participants responded to the open-ended question at the end of the survey asking them what they thought made it hard or easy to follow a meat-free diet during Veganuary. Thematic analysis of these responses resulted in three major themes (see Table 5): “psychological factors,” “tools and resources,” and “social influence.” The former two themes were factors that were perceived as both helping to succeed or be unsupportive of the meat-free diet. For example, in the theme of “tools and resources,” some participants mentioned good availability of vegan products in their local supermarkets as being a helping factor, while for others, a lack of vegan/vegetarian alternatives was the reason for lapsing during Veganuary. The third theme, “social influence,” contained only quotes from participants who had eaten some meat during Veganuary. The prevailing motif here was that a vegan/vegetarian diet was too incompatible with the participant's household diet, so that separate meals would have to be prepared, which was often seen as too much effort or too time-consuming. No participants mentioned other people as a source of support or a helpful factor for sticking to a meat-free diet.

**Table 5:** Results from thematic analysis of challenging and helpful factors in maintaining a meat-free diet during Veganuary.

**Theme**		**No of meat-free (MF) and lapsed (L) responses**	**Exemplar quotations from meat-free (MF) and lapsed (L) participants (typos corrected)**
**Psychological factors:** Responses in this theme mention internal factors, such as self-control, motivation, and, in the case of lapsed respondents (not being able to overcome) habits.		MF: 3	*I did not think about what I was missing out on*
		L: 11	*I didnt have enough self-determination/perseverance to not eat meat*
**Tools and resources:** These were external factors of a very practical nature and were divided into two sub-themes:	**Vegan option availability:** Responses that mention availability of vegan options at home and in supermarkets.	MF: 8	*It was helpful that a lot of the supermarkets were doing new products and launches for veganuary, so there was more choice than usual, but being under lockdown I couldn't go to the ones out of my local area*.
		L: 4	*I went to the shops early January and there wasn't a lot of veg options—no fresh veg, I bought vegan food but decided easier to stick to meat and cheaper also*
	**Information/knowledge:** Having or lacking information on vegan recipes, meal planning or the benefits of a vegan diet.	MF: 3	*A really good vegetarian recipe book by Nigel Slater, being intrigued by different, appealing recipies encouraged me to enjoy eating vegetarian food and helped me stick to eating no meat*
		L: 5	*I wasn't getting enough protein and didn't know what to replace it with*
**Social influence:** Exclusively mentioned by lapsed participants, this theme focused on the influence of other people, usually in the household, including family or a partner, who made it impossible or inconvenient to follow a vegan diet		L: 12	*I ended up eating meat as my partner wasn't willing to give it up fully, most meals I cooked myself an alternative however for some meals it was a lot of effort and easier to cook one meal for us both*

Findings from the “psychological factors” theme which contained quotes from 14 participants (Table 5), extended the quantitative results from this study. Craving or temptation was often mentioned by participants that had lapsed to eating meat, perhaps implying lack of self-control. Interestingly, none of the quotes from successfully meat-free participants in this theme directly mentioned strong self-control as a reason for their success, but they did mention a lack of temptation resulting from some sort of cognitive effort directed at pro-active strategies to avoid temptation, for example: ”*I did not think about what I was missing out on”* (P33) or ”*I think finding lots of different recipes and bulk buying vegetables and protein alternatives meant that I was not tempted to eat a meat based diet”* (P40). This suggests these people directed their thoughts and actions toward pursuing a vegetarian diet, rather than toward resisting the temptation of eating meat. Only one participant in this theme mentioned a disgust evoking experience: ”*A video I watched where the muscles of a piece of meat contracted when cut into.”* (P2).

All raw data are available at: https://osf.io/vkcef/?view_only=aea15b1ad2e44e899191e2699161894b.

## Discussion

The present study aimed to investigate the causal mechanism that may link meat disgust and meat avoidance. We reviewed evidence for the different options of a causal link between the two variables and tested one of these by conducting a study that observed changes in meat disgust in meat eaters that attempted to eat a meat-free diet for 1 month. This approach can be understood as a “quasi-experiment” where participants self-selected the manipulation of a meat-free (or meat-reduced) diet but no manipulation of meat disgust other than this diet change took place.

In order to validate this study, we aimed to reproduce baseline findings from Becker and Lawrence (14) that found a negative association of meat disgust with meat intake in a much larger (combined omnivore and flexitarian *n* = 605) sample. Overall, H1 (meat consumption will be negatively associated with meat disgust at baseline) could partially be accepted as explicit meat disgust negatively predicted meat intake at baseline, before the Veganuary period. However, the effect was much larger here (β = −0.773, *p* < 0.001, *n* = 40), than the effects found in Becker and Lawrence (14) (in omnivores β = −0.190, *p* < 0.001, *n* = 402 and in flexitarians β = −0.349, *n* = 203, *p* < 0.001). One possible explanation for this is that the average baseline levels of explicit meat disgust were different in the two studies: in this sample of omnivores and flexitarians they were higher [*M*(40) = 44.64, *SD* = 24.11] than in Becker and Lawrence's (14) combined omnivore and flexitarian sample [*M*_(605)_ = 24.99, *SD* = 19.06]. This may simply highlight the fact that Veganuary participants are a selective sample with more flexitarian/vegetarian properties (e.g., increased meat disgust). We do not yet know whether or how the effect of meat disgust on meat intake changes at different levels of meat disgust. While this difference in effect size could point toward a non-linear relationship where the effect grows disproportionately to the level of meat disgust, drawing this conclusion from a comparison of two different samples (that are bound to be different in several other ways) would be premature. It does however highlight an interesting focus for further research.

The negative effect of implicit meat disgust on meat intake that Becker and Lawrence (14) found in their flexitarian sub-sample was not reproduced in this study. Instead, implicit meat disgust had an unexpected weakly *positive* effect on meat intake at baseline, whereas the explicit measure of meat disgust had a negative effect, as would be expected. This suggests that our two measures of meat disgust (explicit and implicit), which were not significantly correlated with one another here, tap into different underlying mechanisms, as many others have suggested (48–50). Due to the current limited sample size and inconsistency with our previous findings, we are inclined to trust the result from our previous study with a much larger size. It would be important to replicate the positive association between implicit meat disgust and meat intake in larger samples, perhaps using other measures of implicit attitudes (such as affective priming) in addition to the IAT, before interpreting this effect.

For the main investigation of the causal relationship of meat disgust and meat avoidance, we hypothesized that any meat disgust increases at follow-up would be associated with meat reductions during Veganuary (H2). As before, this expectation was confirmed for explicit, but not for implicit meat disgust, from simple bivariate correlations of change scores. This may be because implicit attitudes tend to change on different time scales (51–54), or because our study was not powerful enough to detect a smaller effect, as our *post-hoc* power analysis suggests. The effect on explicit meat disgust was further confirmed in a regression model predicting changes in this variable: changes in meat intake during Veganuary were most predictive of changes in meat disgust in the expected direction—the more people reduced their meat intake relative to their baseline meat intake, the more their feelings of disgust toward meat grew. An additional predictor of increases in meat disgust was cognitive restraint. A link between heightened restraint and increased disgust toward food or dietary outcomes that people are trying to avoid has been shown by other researchers but so far only in weight loss/obesity-related studies where high cognitive restraint was linked with disgust toward high-calorie food and/or obese body shapes (55–57). Our finding of an association between restraint and disgust toward meat in a sample of people trying to avoid meat presents a novel and interesting expansion of these previous studies. However, the key finding of this study remains that short-term reductions in meat intake can have a powerful effect on increases in meat disgust.

A recent study by Piazza et al. (35) also followed participants during a 28-day meat-free pledge (compared to a non-pledging control group) and measured meat intake and several other factors before, during, and after the pledge period. While the focus of that study was not on disgust, they did measure the effect that attempting a meat-free diet had on meat cravings and found that participants who pledged to not eat meat experienced more meat cravings during the study period than participants in a control condition. Notably, in Piazza et al.'s study (35) participants were randomized to the pledge or control condition and did not self-select to attempt a meat-free diet as was the case in our study—this may limit our findings as the development of meat disgust after only 1 month of meat avoidance may only apply to people who are already motivated to reduce their meat intake. Interestingly, in Piazza et al.'s study (35), omnivores who expressed more “conflictedness” about meat consumption at baseline (e.g., agreeing that eating meat is unethical or unhealthy) were less likely to experience cravings during the period of meat avoidance. This finding may link to the study by Feinberg et al. (21) discussed in the literature review, as “conflictedness” could be seen as an early stage of moralization occurring before or in absence of a transition to vegetarianism. Perhaps our sample of self-selecting Veganuary participants is more akin to Piazza et al.'s (35) more conflicted omnivores which might also help explain the elevated levels of baseline meat disgust in this study as compared to our previous study (14). Additionally, and somewhat counterintuitively, meat cravings can co-occur with meat disgust as previous qualitative studies in vegetarians have reported (8, 9). Meat eaters' conflicted and ambivalent feelings about meat are well-documented, but this conflict usually focuses on the moral athletics that people have to engage in when they simultaneously love and eat animals, known as the meat paradox (58–61). Perhaps this inner conflict also expands to simultaneously experiencing meat cravings and meat disgust, which could be an interesting focus for future research.

The findings reported here (both our own and those of others) have some implications for our understanding of the development of meat disgust. Previous experimental research has shown that increasing disgust toward meat leads to reduced consumption, or willingness to eat meat (24, 25, 33, 34). This may seem obvious but some of this research (24, 25) suggests that only animal (and not plant) source foods can obtain a disgust status. Those findings cannot be explained by the moralization theory proposed by Rozin et al. (17) as discussed in the literature review. Tybur et al. (26) have theorized that because meat poses a greater pathogen threat than plants (24, 62), it may more readily be imbued with disgusting properties and can serve as a pathogen cue. Many other natural stimuli are “prepared” for disgust or fear responses (31, 63) through evolution and this theory can explain why a disgust response to meat (but not to plants) is so easily learned, as demonstrated in evaluative conditioning experiments (24). Our findings demonstrate that disgust to meat can be increased without a deliberately disgust-evoking intervention, simply by avoiding or reducing meat intake for only 1 month. It is still possible that disgust-learning has taken place during the month of meat avoidance/reduction, and one participant did mention a disgusting experience with meat that helped them avoid it, as mentioned above. However, other research demonstrates that people can express disgust to meat they have never had any experience with (25) which is not easily explained *via* preparedness of meat for disgust learning or by moralization.

Therefore, we would like to present a novel theory, building on Tybur et al.'s (26) idea that meat can serve as a pathogen cue. Rather than meat disgust being easily acquired through associative learning processes (perhaps due to preparedness of meat to be viewed as disgusting), we propose that humans may have evolved a blanket disgust response to all meat. This disgust may be the default response when novel meat is encountered [or even when familiar meat is presented in an unfamiliar way (34)] but can easily be suppressed to certain meats, probably aided by social norms of what is culturally acceptable and as a result of positive experiences of eating meat. However, when meat consumption reduces or stops, suppression of meat disgust is no longer necessary, and therefore an increase is seen. A brief look into our evolutionary history could help explain why such a “default disgust” response to meat may have been adaptive to early humans: Compared to our primate ancestors, early humans distinguished themselves (among other things) by developing a much higher meat intake (64) shifting to routinely consuming meat, utilizing it as a major energy and protein source (65). This dietary shift, while beneficial in many ways (66) also exposed humans to an increased risk of pathogen contamination through meat and coincided with the evolution of a human-specific tape worm (67, 68). Early humans in turn needed a new strategy to navigate the pathogen threat of their new diet. Disgust may have evolved as an adaptation to counteract increased meat appetites [much like food neophobia and sensation seeking traits counteract each other in omnivorous animals to balance the threats and benefits that novel foods generally pose (69)]. This is particularly plausible when considering disgust's widely accepted function as a pathogen avoidance mechanism, which is why many of its elicitors (e.g., body fluids, cockroaches, rotten food, sick people, etc.) are pathogen vectors (27–30). Additionally, disgust is highly plastic and can be rapidly acquired, for instance after an episode of food poisoning (70, 71) and suppressed, for instance during sexual arousal (72, 73), or in times of food scarcity (38). A blanket disgust response to all meat that can be downregulated or suppressed for certain exceptions while they are safe to eat seems a tenable theory that is worthy of further investigation.

A secondary focus of this study lay on identifying factors that can act as barriers or facilitators of a meat-free diet. Our statistical analyses did not find any predictors of successful meat reduction during Veganuary (other than baseline meat intake which was controlled for rather than being a predictor of interest). The thematic analysis of participants' comments on what had made it hard or easy to stick to a meat-free diet during Veganuary confirmed that external factors (themes “tools and resources” and “social influence”) seemed to have more of an impact (i.e., were more commonly mentioned) than psychological factors that were measured quantitatively in this study. These findings align well with previous research on barriers and facilitators of meat-free diets [see Graca et al. (74) for a review that places these factors within the COM-B model of behavior]. This suggests that the transition to a meat-free diet (or short-term reduction of meat consumption) can best be aided by removing practical barriers like low availability of plant-based products and lacking knowledge around vegan cooking and perhaps by involving significant others in the transition process. Only one participant mentioned a disgust eliciting video as helping them to avoid meat. This interesting case may be an example of how meat disgust may be expressed suddenly as a result of a disgusting experience rather than appearing more slowly after meat consumption has stopped.

Based on the key limitations of this study [as mentioned above, the low sample size, the special traits of this particular sample (high baseline meat disgust), and the difficulties interpreting the unexpected IAT results], we make the following recommendations for future studies in this area: (i) A larger sample more representative of the general population should be recruited and then randomly assigned (to avoid self-selection bias) to conditions, with meat avoidance as the intervention in the active condition [as in Piazza et al. (35)]; (ii) It would be interesting to measure both “true, underlying” (unsuppressed) disgust and more typical explicit disgust toward meat using comparable measures. For example, VAS ratings (like the ones used here for explicit meat disgust) could be used, with the meat stimuli being of unfamiliar and familiar meat. Pliner and Pelchat (25) have already shown that the simple labeling of meat as familiar or unfamiliar can differentiate between appetite and disgust. It seems logical that an individual's “true” basic level of meat disgust would be expressed to any unfamiliar meat stimulus where no suppression of meat disgust has occurred. Taking VAS ratings from familiar and unfamiliar meat might therefore be a simple, more valid and comparable method of measuring basic and suppressed (rather than implicit and explicit) meat disgust and would further our understanding of this dissociation.

## Conclusion

Very few studies have directly observed attitudinal changes during transitions from an omnivorous to a meat-free or meat-reduced diet (35, 75), and this study adds novel insights to this field by focusing on the role of disgust toward meat. Our findings show that after avoiding or reducing meat intake for only 1 month participants showed an increase in explicit ratings of meat disgust. Further research will help to clarify the underlying mechanisms, e.g., moralization, preparedness for meat as disgust stimulus, or lack of suppression of feelings of disgust, which will contribute to theoretical accounts of meat-eating. Meat disgust may be seen as an interesting extension of the meat paradox, which is usually investigated as a moral dilemma of simultaneously caring for animals and eating them. It seems that in addition, people may also be simultaneously be disgusted by meat and enjoying meat. Both of these paradoxes can be resolved by psychological acrobatics (e.g., denying animal suffering for farm animals, but not for pets, or suppressing meat disgust to the meat of a cow but not to that of a horse) or simply by ceasing to eat meat. Future disgust-based interventions to reduce meat intake should also take into account that people might only need to be reminded of their disgust for meat, rather than having to learn to find meat disgusting.

## Data availability statement

The datasets presented in this study can be found in online repositories. The names of the repository/repositories and accession number(s) can be found below: https://osf.io/vkcef/?view_only=aea15b1ad2e44e899191e2699161894b.

## Ethics statement

The studies involving human participants were reviewed and approved by University of Exeter Psychology Ethics Committee. The patients/participants provided their written informed consent to participate in this study.

## Author contributions

EB contributed to all aspects of this study and prepared the manuscript. SK contributed to participant recruitment, data processing, analysis, and wrote sections of the manuscript. MA contributed to data analysis and interpretation. NL contributed to design and conception of the study, data interpretation, and substantial revisions of the manuscript. All authors contributed to manuscript revision, read, and approved the submitted version.

## Funding

This work was supported by a PhD grant to the EB from the University of Exeter Psychology Department.

## Conflict of interest

The authors declare that the research was conducted in the absence of any commercial or financial relationships that could be construed as a potential conflict of interest.

## Publisher's note

All claims expressed in this article are solely those of the authors and do not necessarily represent those of their affiliated organizations, or those of the publisher, the editors and the reviewers. Any product that may be evaluated in this article, or claim that may be made by its manufacturer, is not guaranteed or endorsed by the publisher.
