# Supplementary material for: The relationship between meat disgust and meat avoidance—A chicken-and-egg problem
Source: Front Nutr. 2022 Sep 2;9:958248. doi: 10.3389/fnut.2022.958248 (PMC9479216; doi:10.3389/fnut.2022.958248)
Supplement: Supplementary file 1 [file Data_Sheet_1.docx]

Supplementary Material


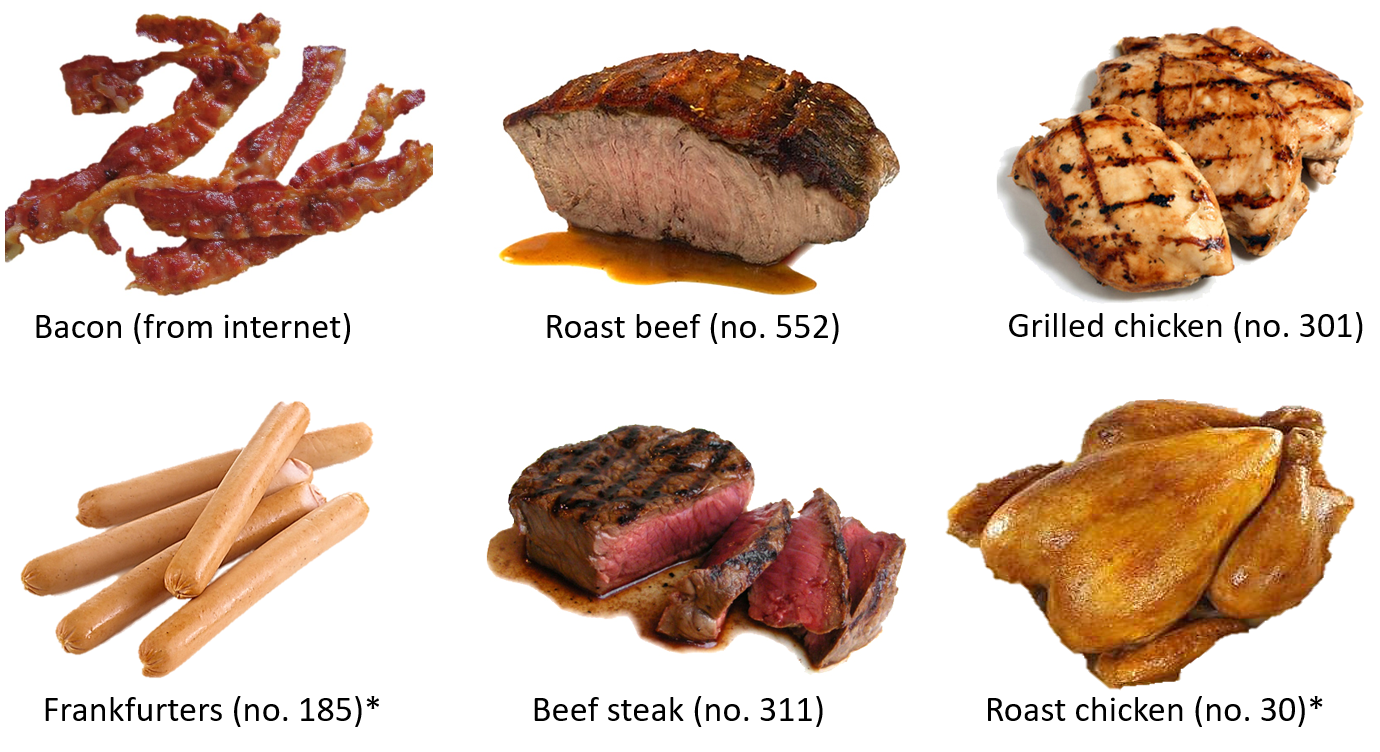
**Figure S1:** Meat images used in both baseline and follow-up surveys. An image of bacon was not available from the Food-pics data base, so this was taken from the internet as it seemed a more culturally appropriate representation of pork than other pork images available in Food-pics (Blechert et al., 2014). All other images are from Food-pics database (numbers in parentheses refer to the image numbers in the Food-pics database). Asterisks indicate pictures that were edited to remove other food or backgrounds that were part of the original images in Food-pics (for example the roast chicken was surrounded by chips).

**Table S1.** Coefficients from multiple regression on baseline meat intake with four predictors.

|  |  | 95% CI | |  |
| --- | --- | --- | --- | --- |
|  | β | lower | upper | p |
| *Disgust Sensitivity* | 0.188 | -0.096 | 0.472 | 0.188 |
| *Cognitive Restraint* | 0.012 | -0.258 | 0.282 | 0.930 |
| *Implicit meat disgust (T1)* | 0.267 | -0.005 | 0.538 | 0.054 |
| *Explicit meat disgust (T1)* | **-0.717** | -1.003 | -0.430 | <.001 |

Note. n = 40, R^2^ = 0.428, R^2^_adj_ = 0.363

**Table S2.** Coefficients from multiple regression on meat intake during Veganuary using emotional eating instead of cognitive restraint as predictor.

|  |  | 95% CI | |  |
| --- | --- | --- | --- | --- |
|  | β | lower | upper | p |
| *Meat intake (T1)* | **0.646** | 0.299 | 0.994 | 0.001 |
| *Explicit meat disgust (T1)* | 0.248 | -0.114 | 0.610 | 0.173 |
| *Implicit meat disgust (T1)* | 0.003 | -0.291 | 0.298 | 0.982 |
| *Emotional eating* | **0.350** | 0.079 | 0.621 | 0.013 |

Note. n = 40, R^2^ = 0.387, R^2^_adj_ = 0.317

**Table S3.** Coefficients from multiple regression on meat intake during Veganuary using uncontrolled eating instead of cognitive restraint as predictor.

|  |  | 95% CI | |  |
| --- | --- | --- | --- | --- |
|  | β | lower | upper | p |
| *Meat intake (T1)* | **0.588** | 0.211 | 0.965 | 0.003 |
| *Explicit meat disgust (T1)* | 0.215 | -0.181 | 0.611 | 0.278 |
| *Implicit meat disgust (T1)* | -0.008 | -0.335 | 0.319 | 0.961 |
| *Uncontrolled eating* | 0.103 | -0.206 | 0.412 | 0.503 |

Note. n = 40, R^2^ =.276, R^2^_adj_ = .193
